# Ischemic aetiology, self-reported frailty, and gender with respect to cognitive impairment in chronic heart failure patients

**DOI:** 10.1186/s12872-016-0349-5

**Published:** 2016-08-30

**Authors:** María J González-Moneo, Gonzalo Sánchez-Benavides, José M Verdu-Rotellar, Mercé Cladellas, Jordi Bruguera, Sonia Quiñones-Ubeda, Cristina Enjuanes, Jordi Peña-Casanova, Josep Comín-Colet

**Affiliations:** 1 Department of Medicine, Universitat Autònoma de Barcelona, Barcelona, Spain; 2 Neurofunctionality of Brain and Language Group, Neurosciences Research Programme, IMIM (Hospital del Mar Medical Research Institute), Barcelona, Spain; 3 Grup de recerca biomedica en malalties del cor GREC (Heart Diseases Biomedical Research Group), IMIM (Hospital del Mar Medical Research Institute), 88, Doctor Aiguader, 08003 Barcelona, Spain; 4 San Martin Primary Care Center, Barcelona, Spain; 5 Jordi Gol University Institute for Research Primary Healthcare, Barcelona, Spain

**Keywords:** Heart failure, Cognitive symptoms, Prevalence, Comorbidity

## Abstract

**Background:**

Decisive information on the parameters involved in cognitive impairment in patients with chronic heart failure is as yet lacking. Our aim was to determine the functional and psychosocial variables related with cognitive impairment using the mini-mental-state examination (MMSE) with age-and education-corrected scores.

**Methods:**

A cohort study of chronic heart failure patients included in an integrated multidisciplinary hospital/primary care program. The MMSE (corrected for age and education in the Spanish population) was administered at enrolment in the program. Analyses were performed in 525 patients. Demographic and clinical variables were collected. Comprehensive assessment included depression (Yesavage), family function (family APGAR), social network (Duke), dependence (Barthel Index), frailty (Barber), and comorbidities. Univariate and multivariate logistic regression were performed to determine the predictors of cognitive impairment.

**Results:**

Cognitive impairment affected 145 patients (27.6 %). Explanatory factors were gender (OR: 2.77 (1.75–4.39) *p <* 0.001), ischemic etiology (OR: 1.99 (1.25–3.17) *p =* 0.004), frailty (OR: 1.58 (0.99 to 2.50, *p =*0.050), albumin > 3.5 (OR: 0.59 (0.35–0.99) *p =* 0.048), and beta-blocker treatment (OR: 0.36 (0.17 to 0.76, *p =* 0.007)). No association was found between cognitive impairment and social support or family function.

**Conclusion:**

The observed prevalence of cognitive impairment using MMSE corrected scores was 27.6 %. A global approach in the management of these patients is needed, especially focusing on women and patients with frailty, low albumin levels, and ischemic aetiology heart failure.

## Background

Cognitive impairment (CI) is particularly common in patients with chronic heart failure (CHF) and has been associated with an increased mortality rate in hospital admissions and worse clinical outcomes [1]. Nevertheless, it is still uncertain when CI should be assessed in common clinical practice, the instruments that should be employed for primary evaluation, and which patients should be included in a more extensive neuropsychological diagnostic battery. Despite interest in the global assessment of CHF there is as yet no decisive information on which factors play a role when deciding whether routine cognition screening in clinical practice would be helpful for an individual patient.

The prevalence of CI depends on the test used and varies widely among studies, ranging from less than 30 % to more than 80 % [2–7]. As cognitive tests are age-and education sensitive [8] the use of a validated screening test in our population would provide valuable information on CI prevalence. The Mini Mental State Examination (MMSE) is a widely used instrument to evaluate cognitive function in CHF patients [9, 10]. It determines global cognitive impairment and is more commonly employed in clinical practice than complex neuropsychological batteries [4, 11].

The present study aims to assess CI prevalence with the MMSE, and identify clinical, psychological, social, family, and frailty-related factors, in a large sample of CHF patients.

## Methods

From 2005 to 2010, 805 consecutive patients were enrolled as a cohort of non-institutionalized patients referred to hospital-primary care integrated multidisciplinary nurse led heart failure program [12]. We present here the baseline assessment. Assessment was conducted at the time of patient inclusion in the heart failure program. Once diagnosis was confirmed on an ambulatory basis, the patient was invited to be included in the heart failure registry. Then, clinical and analytical data were collected, and psychosocial evaluations were performed by hospital nurses specifically trained in heart failure. The evaluation was carried out in a single session, with the cognitive test administered first. The study was conducted in accordance with the Declaration of Helsinki. The study protocol was approved by the local committee of ethics for clinical research and all patients gave written informed consent after recruitment. For inclusion in the study, patients had to be in a stable condition and with a CHF diagnosis of either reduced or preserved ejection fraction, according to the European Society of Cardiology diagnostic criteria [13]. Additionally, inclusion in the study required that patients were able to undergo neuropsychological testing and could communicate adequately in order to follow the tests instructions by themselves. Exclusion criteria for the study were: significant primary valvular disease**,** hemoglobin levels < 8.5 g/dL, clinical signs of fluid overload, pericardial disease, restrictive cardiomyopathy, hypertrophic cardiomyopathy, active malignancy and chronic liver disease. Patients with serious psychiatric illness, unstable CHF, overt cognitive impairment which impeded psychosocial assessment; and those suffering from extra-cardiac disease with a life expectancy of less than 1 year were also excluded. No experimental intervention was performed.

### Clinical variables

At recruitment, peripheral blood samples were taken to measure the usual biological variables needed to perform initial evaluation in heart failure patients. Clinical and demographic information assessed included CHF etiology; New York Heart Association (NYHA) functional class, heart rate, pro-brain natriuretic peptide (Nt proBNP), blood pressure, current medical therapy and the most recent left ventricular ejection fraction (LVEF) evaluation. Recorded comorbidities were current diabetes mellitus, history of stroke or cerebrovascular disease, peripheral arterial disease, chronic kidney disease, anemia, and chronic obstructive pulmonary disease. Once CHF diagnosis had been confirmed on an outpatient basis the patient was invited to be included in the heart failure registry. Clinical and analytical data were then collected, and evaluations were performed by cardiology nurses trained in heart failure management.

### Cognitive functions

**Fig. 1:**
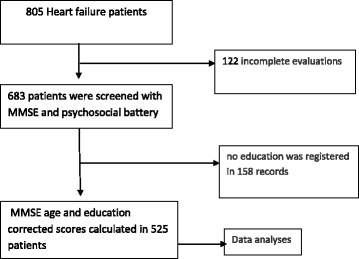
Patient flow showing exclusion criteria

All the patients who agreed to participate underwent a comprehensive psycho-social assessment. From those invited 122 had an incomplete evaluation and were excluded. The MMSEwas completed at baseline in all the 683 remaining patients, either by a neuropsychologist or a specialized nurse who had received specific training. Of those tested, 158 had not education recorded and were excluded. MMSE [14] Spanish validated version (30 items, cognitive impairment scores ≤ 24) [15]) with scores adjusted by age and education was finally calculated in 525 patients (Fig. 1). The MMSE measures overall cognitive impairment, it includes brief assessments of memory, language, praxis, and orientation and it is regarded as the gold standard in cognitive impairment detection [16]. It has been used in heart failure patients [5], it takes approximately 10 min to administer, and It has been found predictor of hospital readmission [3, 15, 17].

Age and education corrected scores were calculated following Blesa MMSE validation in Spanish population. These corrections add or subtract up to 2 points depending on age bands and education level (Table 1). Other corrections have been proposed but external validity of Blesa correction is particularly appropriate as it was carried out in our area. No experimental intervention was performed.

**Table 1:** Correction table of MMSE by age and education. Points added/subtracted from MMSE

Age				
Education (years)		≤50	51–75	>75
	≤8	0	+1	+2
	9–17	−1	0	+1
	>17	−2	−1	0

Based on Blesa [15]

### Psycho-social evaluation

Education was assessed as categorical variable: illiterates, elementary education (4 years of education), high school (up to 9 years), and college (more than 10 years). We recorded marital status, living with a partner, self-administration of medication, and the need for a caregiver.

Dependency was measured by the validated Spanish version of the Barthel test [18] for basic daily activities, in its Spanish validation version; with scores ranging from 0 to 100, those <90indicate dependency. Family function was recorded following The Family APGAR questionnaire in its Spanish form [19], the scale can range from 0 to 10, < 7 represents family dysfunction.

Depression was screened with the short form of the Spanish validated version [20] of the Yesavage Geriatric Depression Scale [21]. Scores > 4 indicate depression.

Social support is positively associated with medication compliance [22–25]. We used The Duke-UNC Functional Social Support Questionnaire [26], an eight-item 3Likert scale in which the higher the average score, the greater the perceived social support. A cut-off of 32 has been proposed in its Spanish version.

Frailty was screened with the self-reported Barber questionnaire [27] in its Spanish validated version [28]. This questionnaire assesses functional status, psychosocial functioning, neurosensory deficits, self-reported health, social support, and previous year hospital admissions. The three major domains in frailty [29]-physical, psychological and social-are represented. It is a 9-item screening tool, easy to understand and self-administer, which identifies individuals who may be at risk of dependency. Any positive item indicates frailty. A cutoff of 2 was applied to increase the positive predictive value of the test since most patients had a recent admission in the previous year.

### Statistical methods

Demographic and clinical data were summarized with basic descriptive statistics in the total cohort. For quantitative variables arithmetic mean (± standard deviation) or median (interquartile range) were calculated as appropriate, and P values derived from a two-sample *t*-test (U-Mann–Whitney tests were used for skewed data). For qualitative variables, percentages within specified groups were calculated and *P* values were derived using Chi [2] tests. All the tests were two-sided; differences were considered significant at the *p <* 0.05 level.

The following clinical variables were dichotomized before entering the linear regression model: age over 65/ below 65, higher education meaning more than 10 years versus less than 10 years of formal education, mild NYHA class I, II versus advanced NYHA III, IV, preserved left ventricle ejection fraction vs. impaired, heart rate over 70 versus HR below 70 as heart rate over 70 is a marker of disease severity and mortality predictor [30]. albumin <3,5 yes/no. NtproBNP was dichotomized over the median as multiple factors influence its values beyond the predictive cut off 1000. Logistic regression models (enter) were generated to explore the relationships between dichotomous and clinical variables. Clinical, functional and biochemical values, echocardiography and co-existing diseases were used as explanatory variables. Univariate analysis was composed of the comparison of cognitive impairment/cognitive normal according to MMSE age-education cut-off scores. Differences between the cognitively normal/impaired groups were calculated using logistic regression model one-to one analyses.

Variables that showed statistical significance (*p <*0.10) in the univariate analysis were included in the multivariable logistic regression (enter method) using MMSE age-and education-corrected scores as the main variable. Three logistic regression models (enter) were completed. Model one included only the clinical factors, model two included psychosocial factors and model three both clinical and psychosocial factors. Results were presented as odds ratios (OR) with 95 % confidence intervals (CI). The Hosmer-Lemeshow chi-square test was used to assess the models’ goodness-of-fit. Various models were developed, including several combinations of adjustment variables. Co-linearity problems were not observed as the change of the standard coefficient errors was not relevant in terms of loss of statistical significance.

All models were carried out in the final patient sample (*n =* 525). SPSS® version 13.0 (IBM, Armonk, NY, USA) was employed for statistical analyses.

## Results

### Cognitive impairment and clinical markers

Of the total sample size (*n =* 525), 145 participants (27.6 %) were affected by CI as determined by MMSE age-and education-corrected scores. There were no differences between participants and non-participants with respect to gender, education level, marital status, CHF etiology, natriuretic peptide levels, ventricular function, cardiovascular risk factors, reactive depression, comorbidity, or need of a caregiver. The former had hypercholesterolemia less frequently (45, 38.5 % vs. 360, 54.1 %, *p =* 0.001) and were more often included in the advanced NYHA functional class (59, 58.4 % vs. 295, 44.3 % *p =* 0.005).

**Fig. 2:**
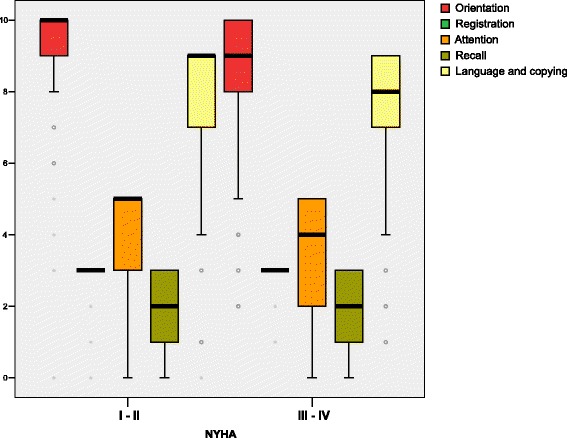
Cognitive impairment in heart failure patients according to Mini-Mental-State Examination sub-items median scores in initial and advanced NHYA functional class. Statistical significance: (Mann-Whinney U): Orientation: *p <*0.001, registration: *p =*0.126, Attention: *p =*0.004, Recall: *p =*0.168, Language and copying: *p =* 0.001. NHYA: New York Heart Association

**Fig. 3:**
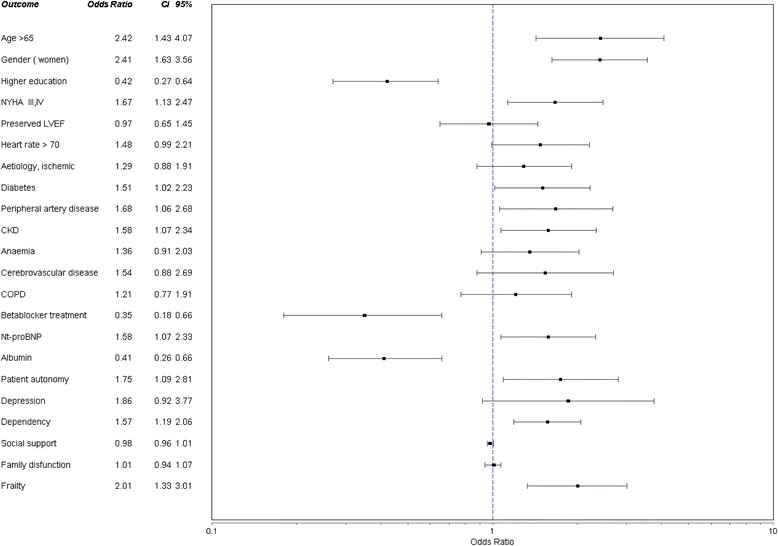
Parameters associated with overt cognitive impairment defined by age and education corrected MMSE scores in univariate analysis with a *P* value < .10. *n =* 525. Risk estimate and confidence limits (CL). Higher education meaning more than 10 years versus less than 10 years of formal education. NYHA: New York Heart Association functional Class. LVEF: left ventricle ejection fraction. DM: diabetes mellitus. CKD: Chronic kidney Disease: eGFR < 60 mL/min/1.73 m^2^. Anemia defined by hemoglobin < 12. COPD: chronic obstructive pulmonary disease. Nt-proBNP: N-terminal pro Brain Natriuretic Peptide hormone. Albumin levels >3.5. Depressive symptoms: measured by Yesavage scores above 5. Family dysfunction: Family Apgar below 7. Social support meaning lack of social support by DUKE-UNC questionnaire < 32. Frailty when Barber questionnaire above 2. Dependency defined by Barthel test <90

The clinically relevant affected MMSE areas in CHF patients with CI were orientation, attention, recall, language, and copying. The worst scores were found in NHYA advanced stages, and only registration was stable in the functional class (Fig. 2). Markers of disease severity such as higher levels of Nt pro-BNP, heart rate > 70, and advanced NHYA functional class were related to CI (Table 2). Mean albumin levels were lower in patients with CI, with a 3.5 cutoff being the most significant; albumin levels > 3.5 were protective, in contrast, albumin levels < 3.5 were related to CI (Fig. 3). Ischemic and hypertensive patients were more impaired than those with heart failure from other causes. Beta-blocker prescription provided a significant reduction in CI (RR: 0.35 (IC95%, 0.18–0.66)) whilst non-use showed an inverse relationship (RR 1.94 (IC 95 %, 1.37–2.74)). No association was found between CI and other drugs commonly used such as angiotensin-converting-enzyme inhibitor and angiotensin receptor blockers

### Gender and education

Patients with CHF and CI impairment were older, had less than 9 years of formal education, and were predominantly female. CI was less common in patients with higher education (defined as more than 9 years of formal education) (Table 2). More men had completed higher education than women [153 (47.8 %) vs. 46 (22.4 %) *p <* 0.001]. CI was more common in women after adjusting by education and NHYA class although the relationship could be moderated by ischemic etiology: CI was more likely in women with ischemic etiology heart failure [OR: 2.303, (95 % IC 1.204–4.405, *p <* 0.001)].

**Table 2:** Demographics and baseline characteristics of Chronic Heart Failure patients according to cognitive function (CI) *n =* 525

	Overall	Normal	Cognitive impairment	*n*	*p* value
*Demographics, n%*	525	380 (72)	145 (28)	525	
*Age, years, mean (SD)*	71 (11)	70 (11)	75 (9)	525	<0.001
*Female gender n (%)*	205 (39)	126 (33)	79 (55)	525	<0.001
*NYHA class n% (I, II / III, IV)*	300 (59) / 211 (41)	229 (62) / 139 (38)	71 (50) / 72 (50)	511	0.007
*Heart rate > =70, bpm, n (%)*	304 (59)	210 (56)	94 (66)	515	0.034
*SBP, mean (SD)*	126 (22)	126 (23)	124 (21)	517	0.324
*Preserved LVEF %, mean (SD)*	180 (34)	131 (34)	49 (34)	525	0.484
*Aetiology of CHF, n%*	382 (73)	268 (71)	114 (79)	520	0.042
*Hypertension n%*	412 (79)	295 (79)	114 (79)	520	0.459
*Diabetes n%*	239 (47)	162 (44)	77 (54)	511	0.023
*Peripheral artery disease n%*	99 (19)	63 (17)	36 (25)	521	0.020
*CKD*	286 (55)	195 (52)	91 (63)	523	0.014
*Anaemia n%*	176 (33)	120 (32)	56 (39)	*525*	0.078
*Cerebrovascular disease n%*	62 (12)	40 (11)	22 (15)	*521*	0.089
*COPD n%*	114 (22)	79 (21)	35 (24)	*517*	0.239
*ACEI or ARBs n%*	426 (82)	311 (83)	115 (80)	*519*	0.243
*Betablockers n%*	482 (92)	358 (95)	124 (86)	*522*	0.001
*MRA n%*	228 (44)	167 (44)	61 (42)	*521*	0.383
*Nt-pro BNP n%*	248 (48)	169 (45)	79 (56)	515	0.014
*Albumin mean (SD)*	4 (0)	4 (0)	3.6 (0)	501	<0.001
*Albumin < 3.5*	97 (19)	57 (16)	40 (28)	501	0.002
*eGFR mean (SD)*	59 (22)	60 (23)	55 (21)	523	<0.017

Cognitive impairment defined by age and education corrected MMSE scores. Data are presented as means ± standard deviation, medians (with interquartil range) or numbers (with percentages) where appropriate. Percentages are rounded. NYHA class: New York Heart Association functional class: LVEF: left ventricle ejection fraction. SBP: systolic blood pressure. CHF: chronic heart failure. Aetiology of CHF: patients with ischemic and hypertensive aetiology versus other causes. CKD: Chronic kidney Disease: eGFR < 60 mL/min/1.73 m^2^. Anaemia was defined as haemoglobin level <12 g/dL in women and <13 gr/dL in men. Cerebrovascular disease defined as. COPD: chronic obstructive pulmonary disease defined following GOLD (Global Initiative of Chronic Obstructive Lung disease) Guidelines. ACEI:angiotensin-converting-enzyme inhibitor. ARBs: Angiotensin II receptor blockers. MRA: Mineral corticoid receptor blockers. Nt-proBNP: N-terminal pro Brain Natriuretic Peptide over 1512 (median scores). eGFR: estimated glomerular filtration rate

### Co-morbidities

We observed a relationship between CI and mean glomerular flow. CI was also more frequent in patients with chronic kidney disease, diabetes, and peripheral vascular disease. Cerebrovascular disease almost reached significance (Table 2) and was included in the final model. Anemia was more common in the CI group but not significant (*p =* 0 .078). Pulmonary obstructive disease was not related with CI in our sample.

### Psychosocial factors

Cognitively impaired patients were more likely in need of a caregiver, and not able to administer treatment by themselves. Decreased CI was related to greater patient autonomy for the management of treatment (Table 3).

Depressive symptoms were more frequent in cognitively impaired patients, almost reaching significance (*p =* 0.064). Lack of social support measured by Duke’s questionnaire was minimal in our sample and non-significant. Less than 15 % of the patients lived in a dysfunctional family, and this factor was un-related to CI.

### Multivariate analyses

Adjusting for the significant factors identified in the unvaried analyses, we performed three different multivariate binary logistic regression models to evaluate (i) clinical factors, (ii) functional ones, and (iii) both. Age-and education-corrected MMSE scores were employed as the main variable in all 525 patients (Table 4). In a clinical model (model 1), patients with overt CI were significantly more likely to be women, and have ischemic etiology and low albumin levels. They were less probably receiving beta-blocker treatment. Dependency and frailty presented a significant association with CI in the functional model (model 2). The presumed association between CI and self-reported frailty almost reached statistical significance after clinical variables were included (*p =* 0.050) (model 3). Gender and ischemic etiology prevailed in the explanatory model when all variables were added. In addition, albumin levels > 3.5 and beta-blocker treatment emerged as potentially protective CI factors.

**Table 3:** Psychosocial, functional and cognitive scores according to cognitive function: *n =* 525

	Overall: 525	Normal *n =* 380	Cognitive impairment *n =* 145	*n*	*p* value
Higher education	199 (38)	164 (43)	35 (24)	*525*	<0.001
Living with someone *n %*	287 (57)	217 (59)	70 (52)	504	0.098
Depressive symptoms *n %*	35 (7)	21 (6)	14 (10)	513	0.064
Dependency *n %*	206 (39)	133 (35)	73 (50)	525	0.001
Need caregiver, *n* (%)	127 (34)	83 (30)	44 (45)	370	0.008
Patient administer treatment *n* (%)	201 (60)	157 (61)	44 (47)	353	0.014
Lack of social support *n* %	41 (8)	27 (7)	14 (10)	515	0.282
Family dysfunction *n* (%)	72 (14)	50 (13)	22 (15)	520	0.325
Frailty *n* (%)	279 (55)	186 (50)	93 (67)	509	0.001
MMSE total age and education corrected scores mean (SD)	25 (4)	27 (2)	20 (3)	525	<0.001
MMSE orientation	9 (1)	10 (1)	8 (2)		<0.001
*n* % task not completed	239 (45)	117 (31)	128 (83)		
MMSE registration	3 (0)	3 (0)	3 (0)		<0.001
*n* % task not completed	27 (5)	11 (3)	15 (10)		
MMSE attention	3 (2)	4 (1)	2 (1)		<0.001
*n* % task not completed	277 (53)	137 (36)	139 (96)		
MMSE recall	2 (1)	2 (1)	1 (1)		<0.001
*n* % task not completed	378 (72)	251 (66)	126 (87)		
MMSE language and copying	8 (2)	8 (1)	6 (2)		<0.001
*n* % task not completed	289 (55)	157 (41)	131 (90)		

Percentages are rounded. Data are presented as numbers (with percentages) or means (standard deviation, SD), where appropriate. n represents the number of patients with variable recorded which were analyzed for each item. Higher education defined as more than 9 years of formal education. Depressive symptoms measured by Yesavage test >5. Dependency in daily living activities measured by Barthel test (total <20/ severe 21–60/ moderate 61–90/slight 91–99 /independence: 100. Social support by The Duke-UNC Functional Social Support Questionnaire (FSSQ), weak social support scores < 32. Family dysfunction by APGAR index < 7; frailty when Barber self-administer questionnaire above 2 (see text) MMSE: mini-mental state examination (Folstein [14]) corrected by age and education (Blesa [15]): cognitive impairment scores ≤24

**Table 4:** Multivariable regression model considering all significative variables (enter)

	Clinical variables Model 1		Psychosocial variables Model 2		Clinical and functional Model 3	
	*OR (95 % CI)*	*p value*	*OR (95 % CI)*	*p value*	*OR (95 % CI)*	*p value*
Gender (women)	2.98 (1.85–4.82)	<0.001			2.77 (1.75–4.39)	<0.001
Ischemic aetiology	1.90 (1.16–3.08)	0.010			1.99 (1.25–3.17)	0.004
Cerebrovascular disease	1.76 (0.94–3.30)	0.079				
Peripheral Arterial disease	1.57 (0.91–2.73)	0.105				
DM	1.15 (0.74–1.79)	0.545				
Heart rate >70	1.44 (0.91–2.27)	0.118				
Albumin > 3.5	0.56 (0.33–0.95)	0.030			0.59 (0.351–0.99)	0.048
*eGFR*	1.01 (0.64–1.58	0.965				
Nt-ProBNP	1.00 (0.63–1.59)	0.990				
Betablockers	0.33 (0.16–0.69)	0.003			0.36 (0.17–0.76)	0.007
Social support			0.83 (0.36–1.94)	0.668		
Frailty			1.58 (1.02–2.46)	0.040	1.58 (0.99–2.50)	0.050
Family dysfunction			1.02 (0.52–1.99)	0.964		
Depressive symptoms			1.72 (0.82–3.63)	0.156		
Dependency			1.65 (1.08–0.53)	0.020	1.30 (0.83–2.05)	0.255

Cognitive impairment defined by MMSE age and education corrected scores

DM: Diabetis Mellitus. eGFR: estimated glomerular filtration rate. Nt-proBNP: N-terminal pro Brain Natriuretic Peptide. Social support measured by The Duke-UNC Functional Social Support Questionnaire (FSSQ); lack of social support scores < 32. Frailty when Barber questionnaire above 2 (see text). Family dysfunction when APGAR index < 7. Depressive symptoms measured by Yesavage test >5. Dependency in daily living activities measured by Barthel test <90. MMSE age and education corrected scores. OR: odds ratio. CI: confidence interval. Hosmer-Lemeshow chi-square test: Model 1 *p =* 0.411; Model 2*p =* 0.766; Model 3:*p =* 0.713. Nagelkerke R Square: Model 1:*R* ^2^ = 0.154; Model 2: *R* ^2^ = 0.052; Model 3: *R* ^2^ = 0.150

## Discussion

### Cognitive impairment

In this single center study we have found that cognitive impairment determined by age-and education-corrected MMSE scores affected 145 of 525 patients (27.6 %), which is consistent with other studies that used this test in patients with heart failure [31]. Nevertheless, other screening methods employed in this type of patients have obtained different figures, as CI prevalence varies according to the test used. Lately, MOCA has emerged as a possible screening tool although it does not take into account the potential false positives that can provide prevalence figures up to 80 % [1]. In a comparative study of the MOCA and MMSE in a small sample of patients, Athilingam et al. (Heart & Lung 2011) found no relationship between clinical parameters of cardiac function and the high rate of CI detected by the MOCA (54 %).

Howkins et al [4] compared the MOCA and MMSE against a neuropsychological battery in 106 patients. They reported adequate sensitivity for both tests (64–70 %) although in terms of specificity, the MMSE was slightly better than the MOCA (70 % vs. 64 %, respectively). This difference in specificity had already been highlighted by Lees et al. [32] in patients with vascular CI.

Both the MOCA and MMSE are clearly influenced by age and education, the former is considered more appropriate in populations with more than 12 years of schooling. The characteristics of the MoCA subtests, which include several complex executive demanding tasks, make it more prone to false positives when it comes to detect cognitive impairment in low educated subjects, making necessary extreme adjustments (up to 4 points in Spanish-speaking individuals; Zhou 2015) [33]. In this regard, we think that MMSE is still useful for its use in low-educated subjects. As a consequence, we employed a validated Spanish MMSE adjusted for age and education which had not only good internal validity but also excellent external validity as it was validated in our area.

Other methods use brief screening tools evaluating only memory, such as the Memory Impairment Screening test (MIS) which in the EFICARE study resulted in 46 % prevalence [34], whilst the MMSE implements more varied subdomains [5–7, 35, 36]. The fact that the prevalence found depends on the test used [5, 37, 38] underlines the importance of using tests validated for the population in focus, with appropriately age-and-education corrected versions. Although there are various tests for mild CI, only MMSE and neuropsychological batteries are recommended for its early detection in Neurology Practice Parameters [39].

The MMSE measures general cognitive functioning, in our study all the sub-items were lower in patients in the advanced functional class, with the exception of registration, which is the ability to acquire the information in the first place, and refers to short-term memory, more influenced by unstable organic conditions [40], and are preserved in many cognitively impaired subjects.

### Clinical markers

We observed that in heart failure patients, ischemic aetiology was linked to CI. In a recent study, lower recall scores in cognitive tests, and cerebral grey matter loss in magnetic resonance images, were reported in patients with ischemic heart disease compared to non-ischemic ones [41]. Mild cerebral ischemia/hypoxia resulting from chronic heart failure could augment the ischemic effect and cause synaptic dysfunction, as recently reported [42]. Hypoperfusion could be an explanation although further research is needed to confirm this possibility.

In concurrence with previous research we found other factors directly linked to heart failure, as such as NHYA advanced class, and Nt proBNP [43]. The relationships were not, however, sustained in a multivariate model. LVEF was not related to CI.

Hypoalbuminemia, previously considered a mortality predictor following myocardial infarction [44], has also been reported to be predictive of 1 year mortality among heart failure patients [45]. Classically linked to frailty, in our sample, albumin levels appeared to be related to CI. We found a cutoff of 3.5 meaningful, as levels < 3.5 were more likely associated to CI. This effect persisted after controlling for renal function and was sustained in a regression model.

In our sample, patients under beta blocker treatment showed less CI after adjusting for functional class, and the effect was maintained after adjusting for multiple factors in a regression model. In the 1990s beta-blocker treatment was associated to cognitive impairment [46, 47]. Nevertheless, recent experimental studies have suggested a protective effect [48]. Such an effect was also observed in a retrospective study of hypertensive patients treated with beta blockers [49] versus other drugs.

### Gender and education

We observed that CI was more frequent in women, who also were less educated. In the Women’s Health Initiative Study [50], self-reported cardiovascular disease was well documented as a factor that incremented the risk of cognitive decline in postmenopausal women, particularly in those with myocardial infarction and vascular disease (HR 2.10; 95 % CI:1.40–3.15), whereas no association was found with self-reported heart failure. Heart failure of ischemic origin was associated to cognitive impairment in women in our study. Further research in women is needed to explore this relationship.

### Co-morbidities

Co-morbidities, including cerebrovascular and peripheral arterial disease [51], must be taken in account in CHF as they have been associated with CI and influence avoidable hospitalization and mortality [52]. A global assessment in CHF patients with co-morbidities illness has been suggested [12]. We found diabetes and peripheral vascular disease to be associated with CI, as previously described [53, 54].

### Psychosocial factors

Our study is aimed at both clinical and psychosocial factors and we identified some that require a global evaluation focused on practice. The identified psychosocial factors included increased patient autonomy (dependency level, self-reported frailty, and need of a caregiver) which was associated with less CI. In addition, social support is positively related to medication compliance [22–24]. In our sample, as previously described in Mediterranean countries [55], almost all the patients had a self-perception of having a healthy social network and satisfactory family function.

Depressive symptoms almost reached signification. Depression was associated with poorer performance on multiple cognitive domains in heart failure in a recent study [53].

Functional limitation has emerged as a predictive value in patients with mild CHF. [56] An elevated relationship between frailty and mortality at 12 months follow-up has been found using the Barthel test, the Yesavage depressive symptoms tests and a geriatric exam [56]. We found a link between self-reported frailty and cognitive function. Frailty and heart failure would share a consistent correlation with some inflammatory biomarkers such as interleukin-6 and C-reactive protein [57]. While there is a lack of consensus on the definition of frailty, assessing it could help tailoring treatment in selected patients. Short screening instruments such as Barber tests could be easy to use in clinical practice. We observed that self-reported frailty remained a stable predictor of cognitive impairment in CHF patients after adjusting for clinical variables.

### Strengths and limitations

The strengths of this study include the wide sample used, the detailed characterization of the profiled patients, and the multi-dimensional assessment including both clinical and psychosocial factors. Our study has some limitations. Its cross-sectional design implies that conclusions should be confirmed through relevant prospective cohort studies. In addition, from those invited to participate, 122 patients had an incomplete evaluation and were excluded. The excluded participants may well have had worse outcomes or have been more impaired as they were in more advanced functional class, resulting in higher figures in our population than reported. Moreover, education was only recorded in 525 patients in a way that could be later analyzed. The unregistered patients lacking education could be also have been more impaired or have had worse CHF markers. It might not be possible, therefore, to extrapolate our results to patients with other education levels. Furthermore, our sample only included Spanish patients so the results may not be applicable to populations from other regions and ethnic groups.

## Conclusions

The observed prevalence of cognitive impairment using MMSE corrected scores was 27.6 %. Screening should focus specially on patients with low albumin levels, frailty, or heart failure of ischemic aetiology, and women in particular. We believe that integrated care improves results in heart failure management and facilitates patient monitoring focused on enhancing quality of life. Almost one in three patients resulted impaired, that justifies the screening in CHF patients as MMSE is a feasible screening instrument, easy to administer on routine settings. Identifying related factors facilitates the selection of patients in which screening would be especially helpful.

## Abbreviations

CHF: Chronic heart failure
CI: Cognitive impairment
LVEF: Left ventricular ejection fraction
MMSE: Mini-mental state examination
NHYA: New York heart association
Nt proBNP: pro-brain natriuretic peptide
